# Retraction: Vesicourachal Diverticulum: A Rare Cause of Recurrent Urinary Tract Infections

**DOI:** 10.7759/cureus.r107

**Published:** 2024-01-25

**Authors:** Mohammed A Alqarni, Rahmah H Kutubkhana, Sara M Alhosami, Faris A Almutairi, Amer M Almutairi, Edris M Alqannad, Mohammed H Almansour, Abrar A Alanazi, Abdulaziz M Althobaiti, Ziyad F Althobaiti, Mohammed A Alshehri, Sulaiman Z Malki, Hussain M Alhasani, Mohammed A Alburi, Faisal Al-Hawaj

**Affiliations:** 1 College of Medicine, King Saud University, Riyadh, SAU; 2 College of Medicine, Batterjee Medical College, Jeddah, SAU; 3 College of Medicine, Ibn Sina National College for Medical Studies, Jeddah, SAU; 4 College of Medicine, King Saud Bin Abdulaziz University for Health Sciences, Riyadh, SAU; 5 General Practice, King Faisal Hospital, Mecca, SAU; 6 College of Medicine, Najran University, Najran, SAU; 7 College of Medicine, Tabuk University, Tabuk, SAU; 8 College of Medicine, Taif University, Taif, SAU; 9 College of Medicine, Taibah University, Medina, SAU; 10 College of Medicine, Umm Al-Qura University, Mecca, SAU; 11 College of Medicine, Vision Colleges, Riyadh, SAU; 12 College of Medicine, Imam Abdulrahman Bin Faisal University, Dammam, SAU

The Editors-in-Chief have retracted this article. Concerns were raised regarding the identity of the authors on this article. Specifically, Faisal Alhaway and Malak Shammari have stated that they were added to this article without their knowledge or approval. The identity of the other authors could also not be verified. As the appropriate authorship of this work cannot be established, the Editors-in-Chief no longer have confidence in the results and conclusions of this article.

